# Sub-groups (profiles) of individuals experiencing post-traumatic growth during the COVID-19 pandemic

**DOI:** 10.3389/fpsyg.2022.969253

**Published:** 2022-09-28

**Authors:** Denise M. Blom, Esther Sulkers, Wendy J. Post, Maya J. Schroevers, Adelita V. Ranchor

**Affiliations:** ^1^Department of Health Psychology, University Medical Center Groningen, University of Groningen, Groningen, Netherlands; ^2^Department of Pedagogy and Educational Sciences, University of Groningen, Groningen, Netherlands

**Keywords:** post-traumatic growth, benefit finding, COVID-19 pandemic, stress appraisal, coping

## Abstract

**Objective:**

Some people experience post-traumatic growth (PTG), entailing positive changes such as a greater appreciation of life following traumatic events. We examined PTG in the context of the negative consequences of the COVID-19 pandemic, notably working from home and social distancing. We aimed to assess whether distinct sub-groups (profiles) of individuals experiencing PTG could be identified by how they appraised and coped with the COVID-19 pandemic.

**Method:**

For this cross-sectional study, we used convenience sampling. In total, 951 participants from the general population completed an online questionnaire with items focusing on primary and secondary appraisal, positive reappraisal, rumination, and coping flexibility. For the latent profile analysis, we selected a sample of 392 individuals who had experienced moderate degrees of pandemic-related PTG, reporting at least two of the 10 positive changes in the PTG Inventory-Short Form.

**Results:**

We identified two distinct profiles among people experiencing PTG. The first was characterised by low levels of primary appraisal and stressfulness and higher levels of secondary appraisal (e.g., resilient group), increased coping flexibility and greater use of positive reappraisal. The second was characterised by higher levels of stressfulness and primary appraisal (e.g., stressed group) and greater use of rumination.

**Conclusion:**

The two sub-groups evidently appraised and coped with the COVID-19 pandemic differently. Therefore, future research should account for these different profiles of people experiencing PTG.

## Introduction

During or after traumatic events, individuals may experience not only negative psychological outcomes, such as depressive symptoms, but also positive changes relating to their personal strength, spiritual beliefs, interpersonal relationships, life priorities and goals and appreciation of life (Tedeschi and Calhoun, 1996, 2004). These positive changes are described as post-traumatic growth (PTG) or benefit finding, which are closely related terms referring to the same phenomenon of positive change occurring in an individual during or after a traumatic event. Previous research findings suggest that many individuals experience PTG not only in the long term but also in the short term and even during stressful circumstances (Wu et al., 2019). For instance, PTG has been reported by 20 to 80% of the samples in previous research within 2 weeks following a sexual assault, within one and 10 months after a cancer diagnosis and within 1 and 3 months after experiencing an earthquake (Frazier et al., 2001; Manne et al., 2004; Marshall et al., 2015). Despite a growing body of research conducted on PTG over the last 20 years, an understanding of why some individuals are more likely than others to experience PTG remains limited. Acquiring insight into who is likely to experience PTG can illuminate ways of increasing PTG, thereby helping individuals to cope with stressful circumstances.

Several theories have attempted to explain the development of PTG. According to Tedeschi and Calhoun (1996, 2004), whose theory is widely accepted, being confronted with a traumatic event may challenge an individual’s fundamental beliefs and assumptions about the world, which may be distressing. This process is thought to induce rumination, which at first is automatic and intrusive, later becoming more deliberate, reflective, and constructive. Together with social sharing, this process may induce the experience of PTG. Other scholars have posited that PTG may be induced when individuals’ understanding about themselves, the world and others is shaken or threatened (Janoff-Bulman, 2004). Thus, in general, theories about PTG posit that it is not so much the event itself that prompts the experience of PTG; rather, it is the emotional and cognitive process of dealing with the event that induces PTG. Likewise, more general models of coping with stress posit that an event is appraised by a person in terms of its impact and that coping strategies (e.g., rumination) are applied to deal with that impact (Folkman, 2010). In turn, appraisal and coping strategies influence an individual’s psychological outcomes, which include PTG.

Previous studies investigated the characteristics proposed by Tedeschi and Calhoun (1996, 2004) along with other relevant factors, such as coping strategies, in an attempt to predict PTG. The only consistent finding is a positive association between positive reappraisal and PTG with larger effect sizes (Helgeson et al., 2006). By contrast, other characteristics, such as sex, age and personality, which are assumed to be predictive of PTG, as theoretically postulated, and various additional characteristics that have been widely examined within the literature, such as stressor characteristics and the severity of the trauma, predicted PTG with low effect sizes. Thus, the findings of studies on the relationships among various characteristics (age, sex, rumination, and coping strategies) are inconsistent, irrespective of the type of traumatic event (Michael and Cooper, 2013; Grace et al., 2015; Casellas-Grau et al., 2017; Bernstein and Pfefferbaum, 2018; Zhai et al., 2019).

One reason for findings of mostly small effect sizes and contrasting results may be that these associations were studied at the group level, whereas there may be individual differences in the predictors of PTG. For instance, it could be argued that for some individuals, the experience of PTG may be the result of ruminative thoughts, whereas for others, a greater perceived impact of the event on their lives could be more strongly associated with PTG. When the results are aggregated at the level of the entire sample, individual-level effects may average out, resulting in small effect sizes or mixed results.

Therefore, rather than examining predictors of PTG at the overall group level, we used a different approach and examined whether there are distinct sub-groups (profiles) of individuals experiencing PTG. An individual-centered approach can be used to identify whether there are individual differences regarding predictors of PTG, thereby indicating that there are multiple pathways for experiencing PTG. We examined such individual PTG profiles in the context of the COVID-19 pandemic. Applying existing theory on PTG (Tedeschi and Calhoun, 1996, 2004; Janoff-Bulman, 2004) and previous empirical findings, we considered socio-demographic characteristics (age and sex), stressor-related characteristics (whether or not the individual belonged to a COVID-19 risk group), subjective appraisal, stressfulness and coping strategies as possible characteristics that differ between people experiencing PTG. We distinguished two types of subjective appraisal. The first is primary appraisal concerning perceived impact of the pandemic and the extent to which the impact is perceived as stressful. The second is secondary appraisal concerning an individual’s belief regarding their ability to cope with the impact.

With respect to the pandemic, recent research indicates that COVID-19 has had a major psychological impact on many people (Salari et al., 2020). Only a few studies have examined the positive outcomes of the pandemic (Zhou et al., 2020; Vazquez et al., 2021). Although the COVID-19 pandemic is still ongoing, there is evidence that PTG was experienced by people right from its onset (Jin et al., 2014; Zhou et al., 2020). For instance, in a study among 2000 Chinese college students, it was found that 67% of students reported PTG, using a cut-off point based on a total score above the 75th percentile as an indication of PTG as suggested by a previous study (Jin et al., 2014). These high prevalence rates of PTG suggest that it is meaningful to examine predictors and individual profiles in PTG in the context of the worldwide COVID-19 pandemic. Previous research has shown that lower levels of PTG were associated with an older age and being female (Zhou et al., 2020). To date, and to the best of our knowledge, no studies have examined the psychological predictors of PTG in the context of COVID-19, including stress appraisals and coping strategies.

## Materials and methods

### Participants

The only requirement for participation in the study was the ability to speak the Dutch language and living in the Netherlands; no other inclusion or exclusion criteria were used. Of the 1,133 participants who opened the online survey, 182 participants were excluded because they only clicked on the survey link, or they did not fill in the sections on demographic information or informed consent. In total, 951 individuals filled in the questionnaire at the baseline measurement at the beginning of the pandemic between 19 April 202 and 21 May 2020; of these participants, 684 completed the questionnaire, and 267 partially completed the questionnaire.

To be able to study PTG and address the research questions, we selected individuals reporting positive changes, using the PTGI-SF. As no clear cutoff exists for the PTGI-SF, we decided to use a cutoff based on the number of changes participants reported. First, we defined a positive change by a score of 3 or higher on an individual item, which indicates the participant experienced the change at least to a moderate degree. Second, a cutoff with higher scores used as an indicator of PTG was based on reporting a change on at least two of the 10 items. We believe this is a relevant indicator of the presence of PTG, while at the same time keeping a sample size with sufficient power. This cutoff yields a sample size of at least 300 participants which is recommended for latent profile analysis (Nylund-Gibson and Choi, 2018). As we realize, this is an arbitrary cutoff point, we conducted a sensitivity analysis, by repeating the analysis among people experiencing at least 1 and at least 3 positive changes to a moderate degree. In total, 438 out of 855 participants who provided data on PTG, achieved scores above this cut-off. Missing values were present for one or more variables in the questionnaires completed by 46 individuals. These individuals’ responses were not therefore included, leaving 392 participants whose responses were analysed.

### Procedure

The study was ethically approved by the Medical Ethics Review Board of the University Medical Center Groningen (UMCG; research register number 202000259). We used baseline measurements obtained from an ongoing longitudinal study on the psychological impact of the COVID-19 pandemic in areas such as well-being, coping strategies, PTG and mindfulness. This study was initiated during the first lockdown in the Netherlands that occurred between 19 April 2020 and 21 May 2020. Various researchers from the Department of Health Psychology at the UMCG invited the general public through Facebook and LinkedIn to participate in the online survey using the Qualtrics software. Followers within their networks could then share the invitations on their own pages. Thus, participants were recruited *via* convenience sampling, which may have induced bias affecting the generalisation of the results. At the beginning of the questionnaire, respondents had to give their consent for their responses to be used anonymously in the analysis.

### Measurements

The following measurements, expressed in the Dutch language, were obtained.

#### PTG

PTG was measured using a validated, abbreviated Dutch version of the PTG Inventory-Short Form (PTGI-SF; Cann et al., 2010). This inventory comprises 10 items requiring respondents to rate their beliefs regarding various positive changes within five domains: relating to others, new possibilities, personal strength, spiritual change and appreciation of life. Sample items are: ‘I appreciate the value of my life more’, and ‘I feel closer to others’. The response scale in the PTGI-SF was adapted to fit the context of the COVID-19 pandemic. The following original response, ‘I did not experience this change as a result of my crisis’, which had the lowest score (0) was adapted as follows: ‘I did not experience this change as a result of the COVID-19 pandemic’. Similarly, the original response, ‘I experienced this change to a very great degree as a result of my crisis’, with the highest score, was adapted as follows: ‘I experienced this change to a very great degree as a result of the COVID-19 pandemic’. A sum score was computed for each participant; a higher score indicated perceiving more positive changes as a result of the COVID-19 pandemic. The internal reliability of the total sample was.88.

#### Perceived impact, stressfulness and secondary appraisal

The items of primary appraisal (perceived impact and stressfulness) and secondary appraisal were based on the SARS Appraisal Inventory (SAI; Cheng et al., 2006). The English questions were adapted to fit the COVID-19 pandemic context and were translated and back translated. Although the measure was not validated, its internal consistency was found to be adequate (0.81–0.88). Perceived impact was assessed in the SAI using multiple items measuring the impact of SARS on multiple life domains (e.g., physical health, career or academic pursuits). In this study, one item was included based on the SAI by assessing the impact of the COVID-19 pandemic on their life. Participants were asked to rate the item using a 5-point Likert scale ranging from 0 (no impact) to 4 (a large impact). If individuals reported an impact (a score ≥1), the stressfulness of this impact was measured using one item, which queried the extent to which this impact of the COVID-19 pandemic on the respondents’ lives was stressful. The response was rated using a scale ranging from 0 (not stressful) to 4 (very stressful).

A single item was used to measure secondary appraisal; the respondent was asked whether they believed that they were capable of coping with the consequences of the COVID-19 pandemic. Like perceived impact and stressfulness, this item was based on the SAI, which assesses an individual’s confidence in coping with the impact of SARS on multiple life domains. Respondents rated the item using a 5-point Likert scale ranging from 0 (not being capable of coping with the consequences) to 4 (being very capable of coping with the consequences of the COVID-19 pandemic).

#### Coping strategies

Positive reappraisal was measured with four items derived from the validated Cognitive Emotion Regulation Questionnaire (CERQ; Garnefski and Spinhoven, 2001). For example, participants were asked to rate their response to ‘I think I can learn something from the situation’. Furthermore, four items were included from the CERQ to measure rumination. An example question is: ‘I often think about how I feel about what I have experienced’. Questions from CERQ were rated on a scale ranging from 0 ([almost] never) to 4 ([almost] always). A sum score was calculated for positive reappraisal and rumination, with higher scores indicating greater use of positive reappraisal and rumination. Internal reliability for the total sample was good for positive reappraisal (0.83) and rumination (0.76).

Coping flexibility was measured with the validated versatility scale of the coping flexibility questionnaire (COFLEX; Vriezekolk et al., 2012). This scale includes nine items, for example, ‘I can easily change my approach if necessary’, which participants rated on a scale ranging from 0 (seldom or never) to 3 (almost always). A sum score was calculated, with higher scores indicating greater coping flexibility. The internal reliability of this scale for the total sample was 0.92.

#### Socio-demographic characteristics

We included sex, age, marital status, education and living situation as socio-demographic characteristics in the study. Respondents could also indicate whether they had children and whether they have a chronic physical illness. Furthermore, they could indicate their employment situation. In case they indicated that they were engaged in wage labour, they could indicate their work situation (i.e., whether or not they worked from home).

#### COVID-19 related characteristics

Respondents could indicate whether or not they believed that they belonged to the COVID-19 risk group. Furthermore, participants could indicate whether they ever perceived COVID-19 like symptoms (either tested or not) or not.

### Statistical analysis plan

Visual inspection of scatter plots of pairs of the included variables revealed no extreme outliers. Therefore, no respondents were omitted from the subsequent analyses. Additionally, the scatter plots revealed no clear profile structure in the data.

Prior to the analysis, the variables were standardised to enable a comparison of the scores for the variables within and between the profiles. To investigate the characteristics of individuals experiencing PTG, we performed a latent profile analysis on the following variables: sex, age, belonging to the risk group, perceived impact, stressfulness, secondary appraisal, coping strategies (rumination and positive reappraisal) and coping flexibility. There were computation issues for the dichotomous variables sex and belonging to the risk group because most participants were female and did not belong to the risk group, resulting in unequal groups. Therefore, separate analyses were performed for each category to explore whether the profiles in each category were similar as they were for the whole group. Because similar results were found for each sex and for belonging (or not) to the risk group, we excluded these categorical variables in the analysis to make the computation and interpretation of the results easier.

The analysis was performed using the ‘tidyLPA’ package in the RStudio software. As there is no consensus on a single fit index for determining the number of profiles, we used the recommendations of Nylund-Gibson and Choi (2018) when examining two fit indices: the Bayesian information criterion (BIC) and the Akaike information criterion (AIC). Although lower scores indicate a more optimal fit for BIC and AIC, these criteria are often associated with a decreasing function. Therefore, they generally yield a large number of profiles leading to a more complex interpretability, while there is only a small gain in the value of the criterium. For this study, the optimal number of profiles was determined at the point where values largely decreased until subsequent profile models showed the ‘elbow point’, indicated by a relatively small decrease.

Subsequently, in the chosen final model, the smallest profile should be larger than 5% of the sample (Hipp and Bauer, 2006) and entropy should preferably be above.80 to indicate greater profile separation (Celeux and Soromenho, 1996). Finally, we considered the interpretation of the profiles when confirming the number of profiles, ensuring that profiles could be clearly distinguished. Accordingly, we plotted the means for characteristics included in the profile models. Up to 10 profiles were considered. The resulting patterns in the final profile model were interpreted using the means and standard deviations of the included variables. Considering that the socio-demographic variables are categorical variables, proportions on characteristics for each profile were given.

Additionally, to test the robustness of the results, we repeated the analyses for a select sample with cutoff points for experiencing one or more positive changes and three or more positive changes to a moderate degree on the PTGI-SF. As no accepted cutoff point for PTG exists, we performed this sensitivity analysis to assess whether the results were in line with the main results and specifically whether profile means were similar.

## Results

### Descriptive characteristics of individuals experiencing higher levels of PTG

Table 1 depicts the descriptive statistics of the respondents. The sample comprised mostly women (84%) with children (66%), a majority of whom were married, in registered partnerships or cohabiting with their partners (69%). A total of 80% of the respondents had completed their higher education and 52% were engaged in wage labour while 60% were working from home during the COVID-19 pandemic. Furthermore, approximately one-fourth (24%) belonged to the COVID-19 risk group, about one-third (32%) had a chronic illness and only a few (13%) had symptoms and thought they had COVID-19 or had tested positive.

**Table 1:** Descriptive statistics (*n* = 392).

Categorical variables	%
Sex (female)	84
**Marital status**	
Married, registered partnership or living together	68
Single, divorced, long-distance relationship or widow	30
Other	2
**Education**	
Lower and middle	20
Higher	78
Other	3
**Living situation**	
Living alone	20
Living with others (e.g., with a partner and/or children)	76
Other	3
Children (yes)	66
Chronic illness (yes)	32
**Employment**	
Wage labour	52
Entrepreneur, retired, incapacitated, unemployed or student	39
Other	10
**Work situation**	
Working from home or sent home	61
Working on-site as usual	23
Other	14
Belonging to a COVID-19 risk group (yes)	22
**COVID-19**	
‘No symptoms’ or ‘I had symptoms, but I do not believe it was COVID-19’	87
‘I had symptoms and I believe this was COVID-19’ or ‘I tested positive’	13
**Continuous variables (range)**	**Mean (SD)**
Age in years (18–89)	50.19 (13.77)
PTG (6–50)	18.71 (8.03)
Perceived impact (0–4)	2.48 (0.95)
Stressfulness (0–4)	1.60 (1.03)
Secondary appraisal (0–4)	2.70 (0.85)
Positive reappraisal (0–16)	9.04 (3.58)
Rumination (0–16)	6.39 (3.15)
Coping flexibility (0–27)	17.28 (5.10)

PTG = post-traumatic growth.

The sample comprised individuals who had experienced at least two positive changes to a moderate degree with reference to the PTGI-SF. Individuals who perceived positive changes had significantly higher scores on positive reappraisal than those who experienced less than two positive changes. Contrary, individuals experiencing at least two positive changes did not differ in terms of their ages, primary appraisals (perceived impact and stressfulness) and secondary appraisals, rumination and coping flexibility compared with those who experienced less than two positive changes to a moderate degree.

Table 2 depicts Pearson correlations among the included variables. Perceived impact was strongly positive in relation to stressfulness (*r* = 0.58) and moderately positive in relation to rumination (*r* = 0.32). Secondary appraisal was moderately negatively correlated with stressfulness (*r* = −0.42), whereas correlations with coping flexibility (*r* = 0.38) and reappraisal (*r* = 0.35) were positive. Finally, stressfulness was moderately positively correlated with rumination (*r* = 0.41), whereas PTG was moderately positively correlated to positive reappraisal (*r* = 0.43).

**Table 2:** Pearson correlations between the variables.

	1	2	3	4	5	6	7
Age	1						
Perceived impact	−0.13^**^	1					
Stressfulness	−0.12^*^	0.58^**^	1				
Secondary appraisal	−0.12^*^	−0.22^*^	−0.42^**^	1			
Positive reappraisal	−0.14^**^	−0.07	−0.20^**^	0.35^**^	1		
Rumination	−0.03	0.32^**^	0.41^**^	−0.20^**^	0.17^**^	1	
Coping flexibility	0.09	−0.05	−0.20^**^	0.38^**^	0.31^**^	−0.04	1
PTG	0.09	−0.07	−0.03	0.12^*^	0.43^**^	0.04	0.17^**^

PTG = post-traumatic growth.

* *p* < 0.05.

** *p* < 0.01.

### Profiles of individuals experiencing higher levels of PTG

#### Number of profiles

Table 3 depicts the fit indices for each profile model. As expected, multiple fit indices indicated different optimal profile models. According to the BIC and AIC, a two-profile model seemed to be the most appropriate model. Specifically, the values at the elbow point showed a relatively small decrease after the two-profile model. Additionally, the BIC value of the measure of fit increased moving from the two-profile model to the three-profile model. Although entropy did not extend beyond the preferred value of 0.80 for any of the profile models, the entropy value (0.79) was best for the two-profile model. Accordingly, the smallest sample size of the profiles, was sufficient in the two-profile model (39% of the sample).

**Table 3:** Model fit indices for LPA.

Profile model	AIC	BIC	Entropy	Minimum N
1	7,766	7,821	1	1
2	7,478	7,565	0.79	0.39
3	7,462	7,581	0.70	0.20
4	7,380	7,531	0.68	0.20
5	7,334	7,517	0.72	0.13
6	7,301	7,515	0.73	0.11
7	7,292	7,539	0.75	0.04
8	7,284	7,562	0.76	0.03
9	7,287	7,597	0.73	0.05
10	7,271	7,613	0.78	0.03

LPA = latent profile analysis; AIC = Akaike information criterion; BIC = Bayesian information criterion.

The two-profile model also seemed appropriate on the basis of our interpretation of the profiles. The two profiles were reproduced in all of the other profile models. Notably, models containing more profiles included additional profiles that were less clearly identifiable than the two dominant profiles. For example, these models were characterised by scores close to the means for all variables or with minor differences compared with those for the two dominant profiles. Considering all of the criteria, the two-profile model appeared to be superior to the one-profile model and was selected as the final model, with, respectively, 238 individuals (60.7%) in profile 1 and 154 individuals in profile 2 (39.3%).

#### The resilient and stressed groups

**Figure 1:**
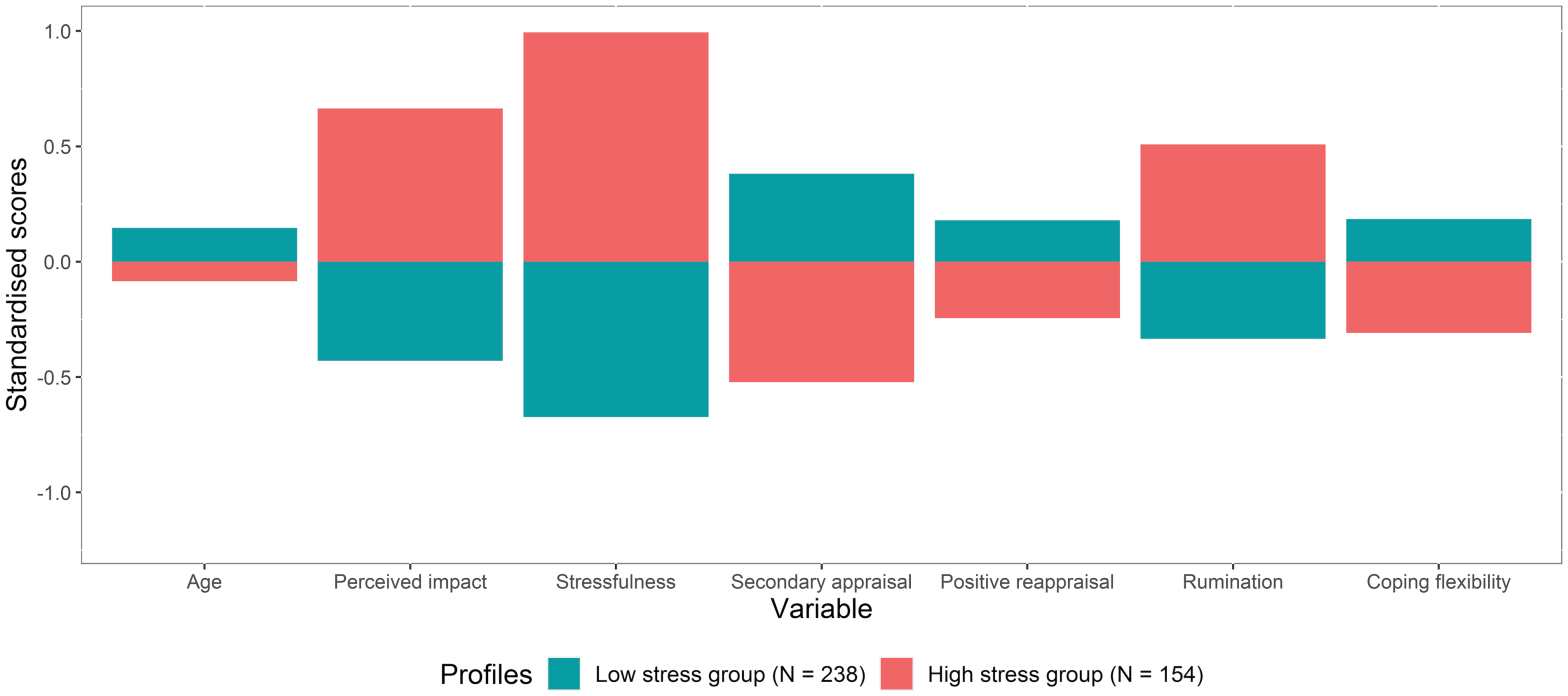
Visualization of the standardised scores of the included variables per profile of the 2-profile model.

Figure 1 visualises the standardised scores of the included variables in the two-profile model. For each included factor on the x-axis, the standardised scores of the included variables can be found on the y-axis for both profiles. Profile 1 (the resilient group) was characterised by lower levels of perceived impact, stressfulness and rumination and higher levels of secondary appraisal, positive reappraisal and coping flexibility. Profile 2 (the stressed group) was characterised in the reverse manner, with higher levels of perceived impact, stressfulness and rumination and lower levels of secondary appraisal, positive reappraisal and coping flexibility. Age was a minor factor influencing the profiles and was relatively similar in both groups.

Table 4 presents the (unstandardised) means and standard deviations of the included variables and socio-demographic characteristics for each profile. The main differences between the profiles were related to the primary and secondary appraisals, stressfulness and rumination. Age, perceived impact, stressfulness, secondary appraisal, positive reappraisal, rumination and coping flexibility differed significantly among the profiles. There were no statistically significant differences among the groups for PTG and the socio-demographic variables with the exception of age. The results of the sensitivity analyses also differentiated these two groups.

**Table 4:** Profile percentages and means (*SD*) per profile.

Categorical variables	Resilient group (*n* = 238)	Stressed group (*n* = 154)
Sex (female)	82	88
**Marital status**		
Married, registered partnership or living together	71	63
Single, divorced, long-distance relationship or widow	28	34
Other	1	3
**Education**		
Lower and middle	16	25
Higher	81	71
Other	2	4
**Living situation**		
Living alone	17	25
Living with others	79	71
Other	3	3
Children (yes)	69	61
Chronic illness (yes)	29	36
**Employment**		
Wage labour	52	52
Entrepreneur, retired, incapacitated, unemployed or student	38	35
Other	10	13
**Work situation**		
Working from home or sent home	69	56
Working on-site as usual	20	27
Other	11	18
Belonging to a COVID-19 risk group (yes)	22	26
**COVID-19**		
‘No symptoms’ or ‘I had symptoms, but I do not believe it was COVID-19’	87	88
‘I had symptoms and I believe this was COVID-19’ or ‘I tested positive’	13	12
**Continuous variables (range)**	**Mean (SD)**	**Mean (SD)**
Age in years (18–89)	51.47 (14.47)	48.21 (12.41)^*^
PTG (6–50)	19.08 (8.04)	18.14 (8.01)
Perceived impact (0–4)	2.06 (0.81)	3.12 (0.78)^*^
Stressfulness (0–4)	0.93 (0.53)	2.65 (0.68)^*^
Secondary appraisal (0–4)	3.00 (0.75)	2.23 (0.77)^*^
Positive reappraisal (0–16)	9.63 (3.35)	8.11 (3.72)^*^
Rumination (0–16)	5.34 (2.72)	8.00 (3.08)^*^
Coping flexibility (0–27)	18.27 (5.08)	15.75 (4.74)^*^

PTG = post-traumatic growth;

* *p* < 0.05, significant difference with resilient group.

## Discussion

Previous studies have overlooked the possibility that people reporting PTG after confrontation with stressful circumstances may differ from each other and show a different profile. Our findings in the context of the COVID-19 pandemic suggest that there are two distinct sub-groups (profiles) of individuals reporting PTG, which differ in terms of their appraisals and coping strategies and, to a lesser extent, age. The so-called resilient group (61% of the sample) perceived a relatively low impact of the pandemic and associated stress on their lives. They were confident in dealing with the COVID-19 pandemic, used more flexible and adaptive coping strategies (i.e., more positive reappraisal and less rumination) and belonged to older age groups than the stressed group. The so-called stressed group (39% of the sample) evidenced the opposite profile, perceiving a relatively high and stressful impact of the COVID-19 pandemic, feeling less confident in dealing with COVID-19 and being more likely to ruminate as a way of coping with stressful situations. They also belonged to younger age groups.

Although the current study did not examine predictors/correlates of PTG, the identification of two distinct profiles within the group of individuals experiencing PTG suggests that there may be different pathways for experiencing PTG. The findings relating to these two groups may explain why previous studies found inconsistent or weak correlates of PTG at the group level. For example, a previous meta-analysis and recent studies indicated that stress predicted PTG inaccurately with a small effect size (Helgeson et al., 2006; Leppma et al., 2018; Seyburn et al., 2019). Our results showed that a perception of higher perceived impact and greater stress associated with the COVID-19 pandemic were only found in one group reporting PTG, whereas the other group perceived lower impact of the pandemic and associated stress. When studying correlates of PTG at the group level, the predictive value of these correlates could average out.

The stressed group that we identified seems more in line with theory stating that PTG is a result of the individual’s struggle and a process of rumination in the aftermath of stressful circumstances (Janoff-Bulman, 2004; Tedeschi and Calhoun, 2004). According to the theory, different types of rumination can be distinguished. The process of PTG and making sense of the circumstances starts with a more intrusive, uncontrollable rumination and continues later with a process of more deliberate rumination (Tedeschi and Calhoun, 2004). Others have made a distinction between rumination as a maladaptive cognitive process (a mode of responding involving perseverative thinking) and reflection (a mode of purposefully processing and thinking about our experiences with the intent of learning something) as a possible adaptive cognitive process (Nolen-Hoeksema et al., 2008). Although the underlying assumption of Tedeschi and Calhoun’s model and this rumination process is that PTG takes time to emerge, previous literature has revealed that PTG is already reported soon after a traumatic event (Jayawickreme and Blackie, 2014). It can be reasoned that for these individuals, the experience of PTG may be a short-term outcome of the perceived impact of the pandemic and the use of rumination as a coping strategy.

Interestingly, we also found a group reporting PTG that perceived the pandemic to have had a low impact on their lives. Members of this group felt confident, were flexible in their coping strategies and made little use of rumination (the resilient group). Even though PTG is widely theorised to occur when an impact or stress associated with an event is experienced, in practice, this may not always be the case. This finding is in line with those of previous studies, which have shown that PTG has been reported by people with and without experiencing a traumatic event and therefore not having necessarily experienced feelings of stress and the impact of the event (Tedeschi and Calhoun, 1996; Taku et al., 2012). Another plausible explanation is that some people may, in general, experience the COVID-19 pandemic less negatively than others. The measures and consequences of the pandemic, such as working from home and having no social obligations, may be perceived more as benefits than as restrictions. Accordingly, individuals could perceive PTG without having experienced the impact of an event and associated stress. The reason may be that the study was conducted during the early stage of the pandemic, whereas its impact could have been perceived later on. Alternatively, it could be argued that PTG reported by the resilient group reflected a more illusory side of PTG, as described in the Janus Face model (Maercker and Zoellner, 2004) and the concept of positive illusions (Taylor, 1983). In this case, perceiving PTG could be a way of coping with the event. Consequently, such individuals may not have perceived the COVID-19 pandemic to have had much impact and associated stress.

Because of the cross-sectional design we could not be sure to what extent the appraisals and coping factors are apparent later in time. Given that our study was conducted at an early stage of the pandemic, longitudinal research is needed to determine whether the two groups continued to be discernible at later stages of the pandemic. Quantitative research could reveal to what extent the two profiles are differentially associated with levels of PTG over time and whether or not the two identified profiles were stable over time. Further research is needed to gain a better understanding of the differences between the two groups of people reporting PTG and to evaluate the theoretical assumptions underlying the interpretations for the groups. This could be done using a qualitative study design that includes interviews conducted with individuals belonging to either group.

From a clinical perspective, more research entailing a person-centred approach is required to study the predictors of PTG. If the finding that individuals experience PTG for different reasons or because of different processes is validated, then interventions could be designed based on a person-centred approach aimed at increasing PTG and helping individuals to find meaning after experiencing a traumatic or stressful event.

### Limitations and strengths of the study

One strength of the study was the novel application of multiple variables within the widely used statistical approach to examine sub-groups of individuals reporting PTG. Furthermore, the variables considered in the study were strongly based on PTG theory. The results revealed one group that was in conformity with theory; in addition, a new pattern of characteristics of people experiencing PTG that has not yet been theorised was also identified. Moreover, the analysis had sufficient power as the sample, comprising more than 300 individuals, was the recommended size (Nylund-Gibson and Choi, 2018). However, there were two limitations relating to the study’s internal validity. The first concerned the arbitrary way of selecting individuals reporting PTG according to their perception of at least two positive changes while the second concerned missing data. These limitations were addressed through additional analyses. Specifically, we conducted analyses of a selected sample of respondents who experienced at least one and at least three positive changes of the PTGI-SF to a moderate degree. Furthermore, to address a small number of missing values, we used multiple imputation to check whether the two profiles emerged in this analysis. These additional analyses also led to the identification of the two distinct profiles, which indicates the robustness of our findings.

Another limitation related to external validity. The sample comprised predominantly well-educated women. Therefore, the results cannot simply be generalised to other populations. The underrepresentation of male participants and individuals of lower education status may be a result of using convenience sampling (Emerson, 2015). Earlier studies conducted during previous pandemics found that less well-educated individuals reported that quarantine had a greater psychological impact compared with better educated individuals (Brooks et al., 2020). This finding could imply that perceptions of the impact of COVID-19 and associated stress were relatively lower within our sample compared to the population including more lower educated males. However, we identified two groups, one of which reported a high impact of COVID-19 and the other a low impact. Moreover, PTG levels were relatively low within this sample. Future research should be conducted including individuals experiencing more PTG, including more males and individuals who are less well-educated in order to confirm these findings. Furthermore, the entropy values of the two-profile model were slightly lower than the preferred value of.80. However, considering the slight deviation from the cut-off and the large number of variables used in this study, the entropy value was considered to be sufficient. The measures for primary and secondary appraisal, which relate to construct validity, were not validated. Even though we used items that were based on an existing appraisal measure, single item measurements cannot be used to average out errors and the specificities of a construct (DeVellis, 2016).

## Conclusion

Previous studies have examined predictors of PTG, such as appraisals and coping strategies, at the group level. In the process, individual or sub-group effects were averaged out, leading to contradictory results. We found that individuals experiencing PTG differed in how they appraised and coped with the impact of the COVID-19 pandemic on their lives. This finding suggests that there are different pathways leading to the experience of PTG.

## Data availability statement

The raw data supporting the conclusions of this article will be made available by the authors, without undue reservation.

## Ethics statement

The studies involving human participants were reviewed and approved by Medical Ethics Review Board of the University Medical Center Groningen. The patients/participants provided their written informed consent to participate in this study.

## Author contributions

DMB drafted the manuscript, carried out data collection and performed data analysis. ES helped carrying out data collection, provided conceptual guidance and helped to draft the manuscript. WP participated in data analysis and helped to draft the manuscript. MS provided conceptual guidance and helped to draft the manuscript. AR provided conceptual guidance and helped to draft the manuscript. All authors read and approved the final manuscript.

## Conflict of interest

The authors declare that the research was conducted in the absence of any commercial or financial relationships that could be construed as a potential conflict of interest.

## Publisher’s note

All claims expressed in this article are solely those of the authors and do not necessarily represent those of their affiliated organizations, or those of the publisher, the editors and the reviewers. Any product that may be evaluated in this article, or claim that may be made by its manufacturer, is not guaranteed or endorsed by the publisher.
